# Physiological and Transcriptional Responses of Sorghum Seedlings Under Alkali Stress

**DOI:** 10.3390/plants14193106

**Published:** 2025-10-09

**Authors:** Xinyu Liu, Bo Wang, Yiyu Zhao, Min Chu, Han Yu, Di Gao, Jiaheng Wang, Ziqi Li, Sibei Liu, Yuhan Li, Yulei Wei, Jinpeng Wei, Jingyu Xu

**Affiliations:** 1 Key Laboratory of Modern Agricultural Cultivation and Crop Germplasm Improvement of Heilongjiang Province, College of Agriculture, Heilongjiang Bayi Agricultural University, Daqing 163319, China; liuxinyu_20@163.com (X.L.); wangboqaq123@163.com (B.W.); zhaoyiyu134340@163.com (Y.Z.); chumin@126.com (M.C.); yuhan09090@163.com (H.Y.); gaodi011022@163.com (D.G.); wangjiaheng202404@163.com (J.W.); LIZIQI716@163.com (Z.L.); liusibei0709@163.com (S.L.); liyuhan011213@163.com (Y.L.); wyl8390@163.com (Y.W.); 2 College of Horticulture and Landscape Architecture, Heilongjiang Bayi Agricultural University, Daqing 163319, China; 3 National Coarse Cereal Engineering Research Center, Daqing 163319, China; 4 Green Food Development Center, Mudanjiang 157000, China

**Keywords:** *Sorghum bicolor* (L.), alkali stress, antioxidant activity, transcriptome, signal transduction

## Abstract

Saline-alkali stress seriously affects the growth and development of crops. *Sorghum bicolor* (L.), a C4 plant, is an important cereal crop in the world, and its growth and geographical distribution are limited by alkali conditions. In this study, sorghum genotypes with different alkaline resistance (alkaline-sensitive Z1 and alkaline-tolerant Z14) were used as experimental materials to explore the effects of alkali on sorghum seedlings. RNA-seq technology was used to examine the differentially expressed genes (DEGs) in alkali-tolerant Z14 to reveal the molecular mechanism of sorghum response to alkali stress. The results showed that plant height, root length, and biomass of both cultivars decreased with time under 80 mM NaHCO_3_ treatment, but Z14 showed better water retention abilities. The photosynthetic fluorescence parameters and chlorophyll content also decreased, but the *Fv*/*Fm*, *ETH*, *ΦPSII*, and chlorophyll content of Z14 were significantly higher than those of Z1. The level of reactive oxygen species (ROS) increased in both sorghum varieties under alkali stress, while the enzyme activities of SOD, POD, CAT, and APX were also significantly increased, especially in Z14, resulting in lower ROS compared with Z1. Transcriptome analysis revealed around 6000 DEGs in Z14 sorghum seedlings under alkali stress, among which 267 DEGs were expressed in all comparison groups. KEGG pathways were enriched in the MAPK signaling pathway, plant hormone signal transduction, and RNA transport. *bHLH*s, *ERF*s, *NAC*s, *MYB*s, and other transcription factor families are actively involved in the response to alkali stress. A large number of genes involved in photosynthesis and the antioxidant system were found to be significantly activated under alkali stress. In the stress signal transduction cascades, Ca^2+^ signal transduction pathway-related genes were activated, about 23 *PP2Cs* in ABA signaling were upregulated, and multiple MAPK and other kinase-related genes were triggered by alkali stress. These findings will help decipher the response mechanism of sorghum to alkali stress and improve its alkali tolerance.

## 1. Introduction

Alkali stress affects plant growth, development, and distribution and can even lead to a decrease in the yield of crops [1]. A variety of factors, such as reduced rainfall, increased land surface temperature, excessive human reclamation, excessive fertilization, unreasonable irrigation, and industrial pollution, lead to soil alkalinity [1,2]. About 9.6 × 10^8^ hectares of agricultural land around the world is affected by soil salinization and alkalization, and this number is still increasing [2]. The area of saline-alkali soil in China has reached 1 × 10^8^ hectares, accounting for about 25% of farmland [2,3]. Soil salinization and alkalization lead to soil degradation, severely damage the ecosystem, limit agricultural productivity, and may even trigger security problems in the human food supply [4,5,6].

Alkali stress persists throughout almost the entire growth and development cycle of plants. Bicarbonate (HCO_3_^−^) and carbonate (CO_3_^2−^) are major contributors to soil alkalinity, and high concentrations of HCO_3_^−^ and CO_3_^2−^ disturb the ionic balance of plant cells, leading to increased osmolality, ROS (reactive oxygen species) accumulation, and oxidative stress [7,8]. Plants mainly mitigate the damage caused by alkali stress by reducing osmotic pressure, oxidative stress, and the ion imbalance induced by alkalinity [7]. For example, under alkali stress, foxtail millet (*Setaria italica* L.) JK3 had higher levels of SOD (superoxide dismutase), POD (peroxidase), and CAT (catalase) enzyme activities and decreased Na+/K+ ratios, with an increase in the content of osmoregulatory substances (soluble protein and proline) so as to relieve oxidative stress and osmotic pressure [9]. The regulatory ability of the root system of Chinese cabbage (*Brassica rapa* L.) with respect to ion absorption was enhanced, and the enzymatic activities of SOD, POD, CAT, and APX (ascorbate peroxidase) increased under alkaline stress [10].

Transcriptomic and metabolomic analysis revealed that a large number of genes related to plant hormone signal transduction, flavonoid biosynthesis, and starch and sugar metabolism were involved in the response of alfalfa (*Medicago sativa* L.) to alkali stress [11]. Comprehensive transcriptome and metabolome analysis revealed that winter rapeseed (*Brassica rapa* L.) improved its alkaline adaptability mainly by activating MAPK signaling pathways and plant hormone signal transduction and regulating starch and sucrose pathways under alkali stress [12]. In addition, the expression of genes encoding ion transport (*HKT1*, *NHX1*, and *NHX2*) was upregulated by alkali stress [13]. Many genes involved in ‘Porphyrin and chlorophyll metabolism’, ‘photosynthesis-antenna proteins’, and’ Photosynthesis’ pathways were downregulated under alkali stress [13]. Through high-throughput discovery of alkaline stress response genes, researchers have tried to improve the adaptability of plants to alkali stress by introducing them into plants using transgenic approaches. Overexpression of *LpMYB4* significantly enhanced the alkali resistance of *Lilium pumilum* DC (*L. pumilum* DC), which was characterized by high chlorophyll content, high antioxidant enzyme activity, and low MDA content [14]. Ketehouli T et al. isolated a soybean CIPK gene (*GmPKS4*), and the overexpression of *GmPKS4* enhanced the removal of reactive oxygen species, the synthesis of osmotic protection solutions, and the transcriptional regulation of stress-related genes in plants under alkali stress [15].

*Sorghum bicolor* (L.), as a C4 plant, is the fifth most important cereal crop in the world and is widely cultivated throughout the world mainly for grain, feed, and biofuel production [16]. Although sorghum has a certain alkaline tolerance, its yield is still significantly reduced under alkali stress [17]. At present, there are only a few reports on alkali stress in plants, and the elucidation of the molecular regulatory network of alkali stress and the mining of alkali tolerance genes are still largely unknown in sorghum. In this study, we selected an alkali-tolerant sorghum germplasm and an alkali-sensitive sorghum germplasm to measure the growth and photosynthetic and physiological parameters of plants under alkali stress, and 80 mM NaHCO_3_ was used to simulate alkali stress. Furthermore, transcriptome analysis of alkali-tolerant Z14 was performed to elucidate the molecular mechanism of alkali tolerance in sorghum. This study provides a theoretical basis for the study of the alkali tolerance mechanism of sorghum and also provides some reference for the breeding of alkali-tolerant sorghum varieties.

## 2. Results

### 2.1. Responses of Sorghum Seedlings of Different Genotypes to Alkaline Stress

**Figure 1:**
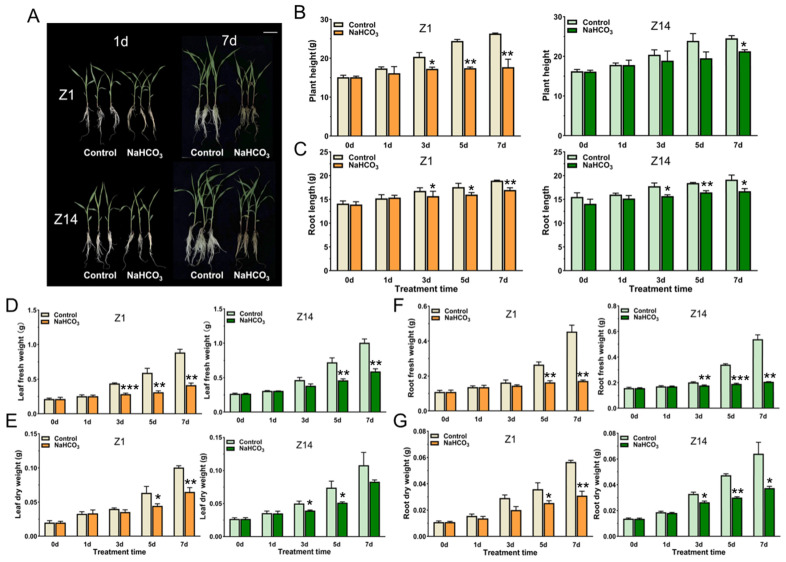
Phenotypic parameters of different sorghum genotypes under 80 mM NaHCO_3_ treatment. (**A**) Phenotypes of sorghum seedlings. (**B**) Plant height. (**C**) Root length. (**D**) Fresh weight of leaves. (**E**) Leaf dry weight. (**F**) Root fresh weight. (**G**) Root dry weight. Content levels are expressed as mean ± standard error (n ≥ 3). Significance levels were calculated via Student’s *t*-test: * *p* < 0.05, ** *p* < 0.01, and *** *p* < 0.001.

To evaluate the effects of alkali stress on the growth of sorghum seedlings, the physiological responses of two different genotypes of sorghum (alkali-sensitive Z1 and alkali-tolerant Z14) to alkaline stress were determined. As shown in Figure 1, compared with the control (without NaHCO_3_ treatment), the growth of Z1 was significantly hindered, the leaves wilted and yellowed, and the damage degree of Z1 was significantly higher than that of Z14 at 7 d of alkali treatment (80 mM NaHCO_3_) (Figure 1A). Under alkali treatment, the plant height (Figure 1B) and root length (Figure 1C) of sorghum seedlings decreased significantly. Compared with the control, at 7 d, the plant height and root length of Z1 and Z14 decreased by 32.78% and 13.32% and 10.39% and 12.72%, respectively. The fresh weight and dry weight of aboveground and underground parts of Z1 and Z14 were significantly reduced under alkali stress, which was most obvious at 7 d. Compared with the control, they were reduced by about 53.33% and 41.22%, 35.60% and 23.01%, 62.22% and 61.75%, and 45.25% and 41.64%, respectively. The decrease in growth metrics was significantly greater in Z1 than in Z14 (Figure 1D–G). The results showed that alkali stress severely affected the growth phenotype of sorghum and inhibited its growth and biomass accumulation.

### 2.2. Effects of Chlorophyll, Carotenoids, and Photosynthetic Fluorescence Parameters of Sorghum Seedlings Under Alkali Stress

**Figure 2:**
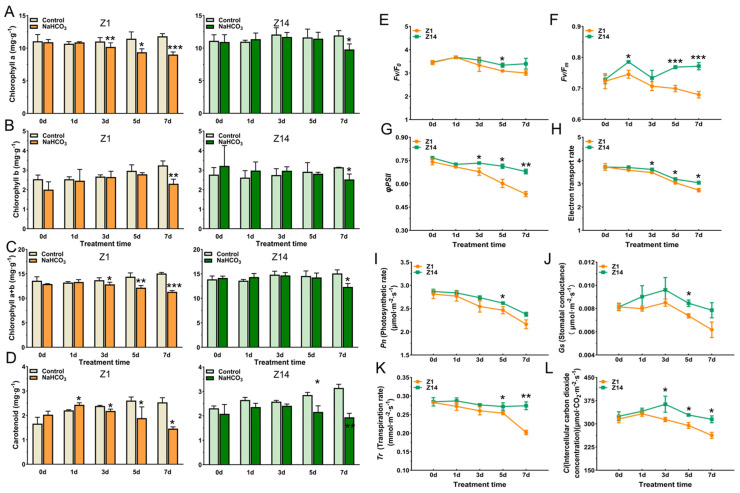
Effects of alkali stress on chlorophyll, carotenoids, and photosynthetic fluorescence parameters of Z1 and Z14. (**A**) Chlorophyll a content. (**B**) Chlorophyll b content. (**C**) Total chlorophyll content (chlorophyll a + b). (**D**) Carotenoid content. (**E**) Maximum light energy conversion potential (*Fv*/*F_0_*). (**F**) Maximum photochemical efficiency of PSII (*Fv*/*Fm*). (**G**) Quantum yield of PSII (*ΦPSII*). (**H**) Electron transport rate (ETR). (**I**) Photosynthetic rate (*Pn*). (**J**) Stomatal conductance (*Gs*). (**K**) Transpiration rate (*Tr*). (**L**) Intercellular carbon dioxide concentration (*Ci*). Significance levels were calculated via Student’s *t*-test: * *p* < 0.05, ** *p* < 0.01, and *** *p* < 0.001.

The parameters of chlorophyll content, photosynthesis, and fluorescence of the third fully expanded leaf were measured to analyze the effects of alkali stress on photosynthesis in sorghum seedlings (Figure 2). From the third day of alkali treatment, Z1 showed a significant difference compared with the control, and the chlorophyll content decreased by about 7.49%, 18.13%, and 23.72% at 3, 5, and 7 d under alkali treatment, respectively (Figure 2A). However, Z14 showed a significant difference only on the 7 d of alkali treatment, and the chlorophyll content decreased by about 18.02% compared with the control (Figure 2A). The content of chlorophyll b in the leaves of the two sorghum varieties was significantly lower than that of the control on the 7 d of treatment, which was reduced by about 29.04% and 19.79%, respectively (Figure 2B). Compared with the control, Z1 showed a significant difference at 3 days and decreased by 6.19%, 15.64%, and 24.86% at 3, 5, and 7 d, respectively (Figure 2C). However, Z14 showed a significant difference only at 7 d compared with the control. The chlorophyll content was reduced by about 18.39% (Figure 2C). Z1 and Z14 carotenoids showed a first increasing and then decreasing trend with an increase in treatment time, and the contents reached the lowest on the 7 d of alkali treatment, which were about 57.54% and 61.74% of the control, respectively (Figure 2D).

The chlorophyll fluorescence parameters reflect the photosynthetic capacity of plants. The photosynthetic fluorescence parameters and gas exchange parameters were measured to analyze the effect of alkali stress on photosynthesis. The chlorophyll fluorescence parameters gradually decreased with the alkali treatment’s time, among which the maximum light energy conversion potential (*Fv*/*F_0_*) of Z1 and Z14 reached a significant difference on the 5th day, and the *Fv*/*F_0_* of Z1 was about 7.38% lower than that of Z14 (Figure 2E). The PSII maximum photochemical efficiency (Fv/Fm) of Z1 and Z14 reached a significant difference at 1, 5, and 7 d, with the Fv/Fm of Z1 being approximately 5.05%, 8.98%, and 11.92% lower than that of Z14, respectively (Figure 2F). The quantum yield (*ΦPSII*) and electron transport rate (ETR) of PSII of Z1 were significantly lower than those of Z14 after 3 days of treatment and were about 7.58%, 15.53%, and 21.35% and 3.78%, 4.69%, and 10.50% lower than those of Z14 at 3, 5, and 7 d of treatment, respectively (Figure 2G,H). Subsequently, we analyzed four additional photosynthesis-related parameters, and the results are shown in Figure 2I–L. *Pn* decreased gradually with the extension of time, and it reached a significant difference on the fifth day of treatment. *Pn* of Z1 was about 5.86% lower than that of Z14 (Figure 2I). *Gs* increased first and then decreased with time. At 5 d, the *Gs* of Z1 was about 12.74% lower than that of Z14 (Figure 2J). A similar trend was observed for *Tr* and *Pn*, with a significant difference in *Tr* between Z1 and Z14 at 5 d and 7 d of alkali stress, which decreased by about 6.50% and 26.31%, respectively, compared with Z14 (Figure 2K). However, *Ci* had the same trend as *Gs*. On the 5th day of alkali treatment, *Ci* of Z1 was significantly reduced by about 10.34% compared with Z14 (Figure 2L). The results showed that alkali stress impaired photosynthetic pigment accumulation and inhibited photosynthesis in sorghum leaves.

### 2.3. Response of the Antioxidant System of Sorghum Seedling Leaves Under Alkali Stress

**Figure 3:**
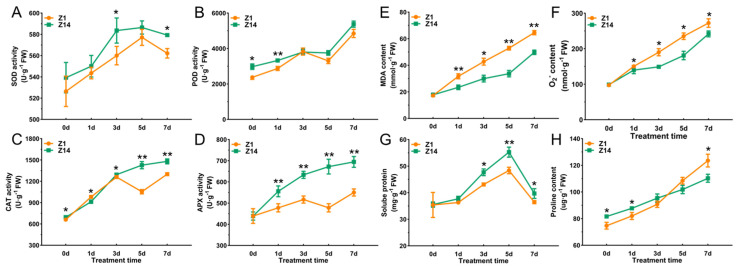
Effects of alkali stress on the antioxidant system of sorghum seedlings under 80 mM NaHCO_3_ treatment. (**A**) SOD activity. (**B**) POD activity. (**C**) CAT activity. (**D**) APX activity. (**E**) Malondialdehyde (MDA) content. (**F**) Superoxide anion (O_2_^−^) content. (**G**) Soluble protein content. (**H**) Proline content. Significance levels were calculated via Student’s *t*-test: * *p* < 0.05, and ** *p* < 0.01.

The activities of antioxidant enzymes (SOD, POD, CAT, and APX) and the contents of osmoregulatory substances reflect the ability of plants to remove ROS and regulate osmotic pressure, while the contents of MDA and O_2_^−^ (superoxide anion) reflect the degree of plant damage. In order to systematically analyze the antioxidant response of sorghum seedling leaves to alkali, multiple physiological indicators related to antioxidant enzymes, membrane damage (MDA and O_2_^−^), and osmoregulatory substances were determined, and the results are shown in Figure 3. Under the 80 mM NaHCO_3_ treatment, the activities of SOD, POD, CAT, and APX in Z1 and Z14 gradually increased with treatment times. Among them, the SOD activity of Z1 and Z14 reached a significant difference at 3 d and 7 d, and the SOD activity of Z14 was 4.21% and 3.05% higher than that of Z1, respectively (Figure 3A). Compared with Z1, the POD activity of Z14 reached a significant difference at 0 d and 1 d of treatment, which was 25.26% and 15.65% higher than that of Z1, respectively (Figure 3B). However, the CAT activity of Z1 was 6.80% higher than that of Z14 at 0 d of alkali treatment, while the CAT activity of Z14 was higher than that of Z1 at the remaining time points (Figure 3C). From the first day of alkali treatment, the APX activity of Z14 was higher than that of Z1, and it was 16.28%, 22.58%, 40.70%, and 26.26% higher than that of Z1 at 1, 3, 5, and 7 d of alkali treatment, respectively (Figure 3D). These results suggest that Z1 and Z14 eliminate ROS accumulation to maintain normal plant growth by enhancing their own antioxidant enzyme activities.

With the extension of stress time, the MDA content and O_2_^−^ content of the two varieties showed a gradually increasing trend, both reaching the peak on the 7 d of treatment. At the 7 d of treatment, MDA content and O_2_^−^ content in Z1 were 22.91% and 19.50% higher than that in Z14 (Figure 3E,F). Among them, the MDA content and O_2_^−^ content of Z1 were significantly higher than those of Z14 except for 0 d (Figure 3E,F). This indicated that the membrane damage was aggravated with time under alkali stress, and the degree of damage was lower in Z14 than in Z1. In addition, the soluble protein contents of Z1 and Z14 increased first and then decreased with the extension of treatment time. After 3, 5, and 7 d of alkali treatment, the soluble protein content of Z1 and Z14 was significantly different, and the soluble protein content of Z14 was 8.72%, 6.68%, and 7.33% higher than that of Z1, respectively (Figure 3G). At the same time, the proline content of the two sorghum varieties gradually increased with the extension of alkali treatment times. Among them, the proline content of Z14 was 8.52% and 6.60% higher than that of Z1 at 0 d and 1 d (Figure 3H).

### 2.4. Differentially Expressed Genes in Leaves of Z14 Sorghum Variety Under Alkali Stress

In order to understand the molecular mechanism of alkali stress response in sorghum seedlings, we used RNA-seq to analyze the differentially expressed genes (DEGs) in the leaves of alkali-tolerant seedlings. As shown in Table S1, a total of 116.38 Gb of filtered data was obtained, and the filtered data were higher than 6.85 Gb for each sample. Analyses of the FPKM density distribution of each sample showed that the gene expression of all samples was distributed between −2.1 ≤ log_10_(FPKM) ≤ 2.4 (Figure S1A). Correlation analysis showed a high degree of agreement between the qRT-PCR and RNA-seq results, with a Pearson correlation coefficient of 0.8248, confirming the reliability of the transcriptome data (Figure S2).

Under the screening criteria of −1 ≤ Log_2_FC ≤ 1, differentially expressed genes (DEGs) were screened from different comparison groups, and the results are shown in Figure S3. Figure S3A shows that SA12h vs. SA0h, SA24h vs. SA0h, and SA72h vs. SA0h (SA, salt-alkaline) resulted in 2619, 450, and 825 upregulated differentially expressed genes and 2707, 300, and 778 downregulated differentially expressed genes, respectively. SA24h vs. SA12h, SA72h vs. SA12h, and SA72h vs. SA24h resulted in 2737, 2621, and 187 upregulated genes and 2847, 3191, and 152 downregulated genes, respectively (Figure S3C). In order to better observe the level of DEGs, we created a volcano plot, as shown in Figure S3C. These results indicated that DEGs responded to alkali stress to varying degrees at different time points.

### 2.5. Enrichment of DEGs in Z14 Sorghum Seedlings Under Alkali Stress

**Figure 4:**
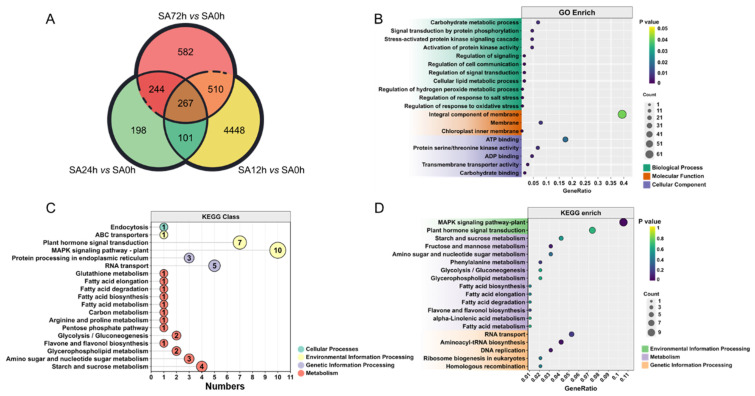
GO and KEGG enrichment of DEGs in Z14 sorghum seedlings under 80 mM NaHCO_3_ treatment. (**A**) Venn diagram analysis of DEGs. (**B**) GO enrich analysis of 267 co-expressed DEGs. (**C**) KEGG classification of 267 co-expressed DEGs. (**D**) KEGG enrichment analysis of 267 co-expressed DEGs.

In order to better explore the differences in the response of Z14 to alkali stress, a Venn diagram was drawn for each comparison group, as shown in Figure 4A. There were 4448 DEGs expressed in the SA12h vs. SA0h comparison group, 198 DEGs expressed in the SA24h vs. SA0h comparison group, and 582 DEGs expressed in the SA72h vs. SA0h comparison group. There were 267 common differentially expressed genes in the SA12h vs. SA0h, SA12h vs. SA0h, and SA72h vs. SA0h comparison groups. These 267 DEGs represent genes with specific high responses at each of the three time points of treatment, 12, 24, and 72 h, indicating that these 267 DEGs are genes that respond to alkali stress.

Subsequently, GO (Gene Ontology) enrichment analysis was performed on these 267 co-expressed DEGs under the screening criteria of *p*-value ≤ 0.05. The results showed that the enrichment results were mainly divided into three categories: biological process, molecular function, and cellular component (Figure 4B and Table S2). In the biological process category, the lower *p*-value comprised signal transduction by protein phosphorylation (GO:0023014), stress-activated protein kinase signaling cascades (GO:0031098), activation of protein kinase activity (GO:0032147), regulation of response to salt stress (GO:1901000), regulation of response to oxidative stress (GO:1902882), etc. (Figure 4B and Table S2). An integral component of the membrane (GO:0016021) was enriched in the molecular function category. Membranes (GO:0016020) and chloroplast inner membranes (GO:0009706) were observed in three pathways (Figure 4B and Table S2). The cellular component category was mainly enriched in five pathways, including the following: ATP binding (GO:0005524), protein serine/threonine kinase activity (GO:0004674), ADP binding (GO:0043531), transmembrane transporter activity (GO:0022857), and carbohydrate binding (GO:0030246). These results indicated that the expression of related genes involved in the above pathways was induced by alkali stress.

In order to better understand the biological functions of common DEGs, KEGG (Kyoto Encyclopedia of Genes and Genomes) classification analysis was performed. As shown in Figure 4C (Table S3), the metabolic pathways involved in 267 common DEGs were mainly divided into four branches, and further statistics were performed for each branch (Figure 4C and Table S3). The MAPK signaling pathway–plant (10), plant hormone signal transduction (7), RNA transport (5), and metabolic pathway (10) were the most frequently annotated genes, followed by aminoacyl-tRNA biosynthesis (4), starch and sucrose metabolism (4), etc. (Figure 4D and Table S4). The results suggest that genes involved in these pathways may be involved in the adaptive regulation of plant alkaloid responses.

### 2.6. Analysis of Differentially Expressed Transcription Factors in Z14 Sorghum Leaves Under Alkali Stress

**Figure 5:**
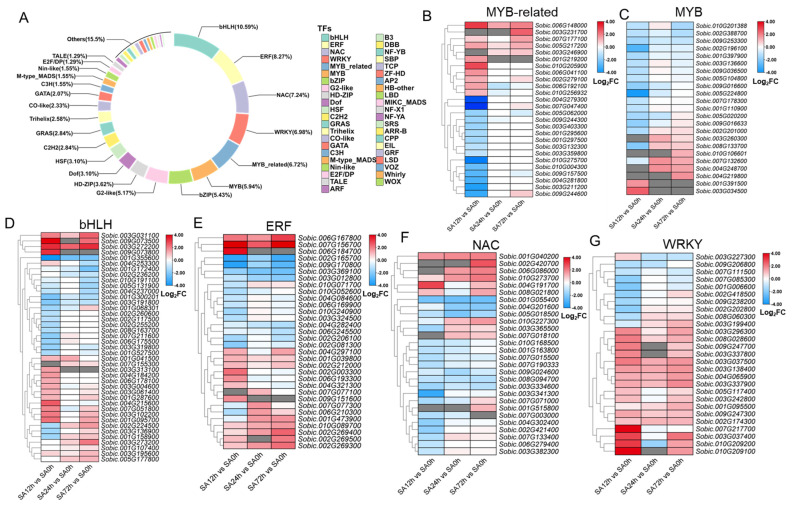
Expression profiles of differential transcription factors in Z14 sorghum seedlings under 80 mM NaHCO_3_ treatment. (**A**) Pie chart of differential transcription factors. (**B**) Heat map of *MYB-related* TF expression. (**C**) Heatmap of *MYB* TF expression. (**D**) Heatmap of *bHLH* TF expression. (**E**) Heatmap of *ERF* TF expression. (**F**) Heatmap of *NAC* TF expression. (**G**) Heatmap of *WRKY* TF expression.

To more clearly define the transcriptional regulation in Z14 leaves under alkali treatment, we performed statistical analysis of differentially expressed transcription factors (TFs) in the transcriptome data (−1 ≤ Log2FC ≤ 1). As shown in Figure 5 and Figure S4, a total of 387 DEGs were found to encode 43 TFs. Among them, transcription factor families included *bHLH* (10.59%), ERF (8.27%), *NAC* (7.24%), *WRKY* (6.98%), and *MYB-related* (6.72%) and *MYB* (5.94%) families, which were significantly representative. In addition, unlisted TF families (fewer than four TFS or less than 1.1% of TFS) were classified as other. Figure 5B–G show that the expression survey of the top six transcription factor families (*MYB-related*, *MYB*, *bHLH*, *ERF*, *NAC,* and *WRKY*) in different comparison groups of alkali treatment shows that most TFs are strongly responsive to alkali stress and upregulated after alkali treatment.

### 2.7. Expression Profiles of DEGs Involved in Physiological Responses in Z14 Sorghum Seedlings Under Alkali Stress

**Figure 6:**
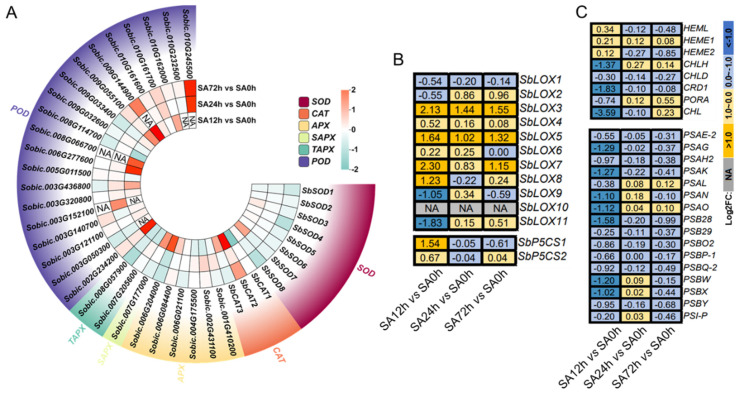
Expression profiles of DEGs involved in physiological responses in Z14 sorghum seedlings under 80 mM NaHCO_3_ treatment. (**A**) Expression levels of *SOD*s, *POD*s, *CAT*s, and *APX*s. (**B**) Expression levels of *LOX*s and *P5CS*s. (**C**) Expression levels of genes involved in chlorophyll synthesis and leaf photosynthesis.

DEGs related to the previously observed physiological phenotype changes were specifically screened out from the transcriptome data, including genes related to antioxidant enzymes, osmotic regulation, membrane damage, chlorophyll metabolism, and plant photosynthesis. As shown in Figure 6, a large number of genes encoding antioxidant enzymes were discovered, including 8 *SbSOD*s, 20 *SbPOD*s, 3 *SbCAT*s, and 9 *SbAPX*s, and some of them were significantly activated under alkali stress (Figure 6A). *SbSOD7* was found to be upregulated in the SA12h vs. SA0h comparison group with a fold change (Log_2_FC) of 1.33. *SbSOD4* and *SbSOD8* were downregulated in the SA72h vs. SA0h comparison group by a fold change of −1.11. The expression fold of the remaining *SbSOD*s was not significant in the SA12h vs. SA0h, SA24h vs. SA0h, and SA72h vs. SA0h comparison groups. However, we found that most *SbPOD*s were upregulated in response to alkali treatment. *SbCAT1* was downregulated in the SA12h vs. SA0h comparison group, while *SbCAT2* and *SbCAT3* were upregulated in the SA12h vs. SA0h comparison group by 3.69 and 1.24 folds, respectively. *SbCAT2* was also upregulated in the SA72h vs. SA0h comparison group by 1.45 folds. Nine genes encoding *APX* were screened, including six cytoplasmic *APXs* and two chloroplast *APX*s, which additionally contained matrix *APX* (*SAPX*) and thylakoid *APX* (*TAPX*). In the SA12h vs. SA0h comparison group, the expression of *Sobic.006G204000* was the highest (2.62 folds), while in the other comparison groups, the *SbAPX* gene was expressed in different degrees.

Subsequently, the expression profiles of *LOX*s, genes related to MDA synthesis, and *P5CS*s, genes related to osmoregulation, were analyzed (Figure 6B). The results showed that the expression levels of most *SbLOX* genes were increased under alkali stress in SA12h vs. SA0h, SA24h vs. SA0h, and SA72h vs. SA0h comparison groups. The expression of the *SbP5CS1* gene was upregulated in the SA12h vs. SA0h comparison group by 1.54 folds. These results indicate that the expression of *SbLOX*s is upregulated and MDA synthesis is accelerated under alkali conditions. However, *SbP5CS1* is upregulated by alkali induction, which promotes the production of osmoregulatory substances to maintain normal osmotic pressure in plants. We further analyzed the genes related to chlorophyll metabolism and photosynthesis, and the results are shown in Figure 6C. The results showed that most genes were downregulated in the SA12h vs. SA0h comparison group. However, no significant gene expression was achieved in the SA24h vs. SA0h and SA72h vs. SA0h comparison groups.

### 2.8. Expression Profiles of DEGs Involved in Stress Signaling in Sorghum Seedlings Z14 Under Alkali Stress

**Figure 7:**
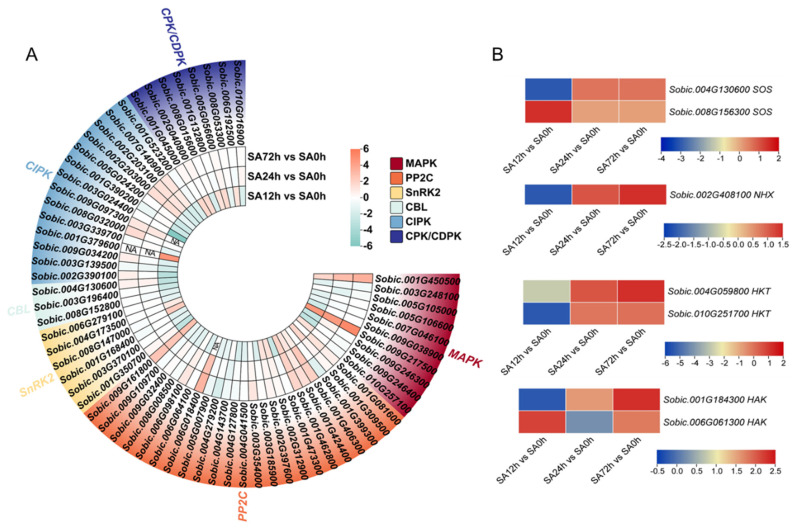
Expression profiles of signal-transduction-related genes and salt-stress-related genes under 80 mM NaHCO_3_ treatment. (**A**) Expression levels of *CPK*s/*CDPK*s, *CIPK*s, *CBL*s, *SnRK2*s, *PP2C*s, and *MAPK*s. (**B**) Expression levels of *SOS*s, *NHX*, *e*s, and *HAK*s.

In this study, KEGG and GO enrichment analysis identified plant signal transduction and the protein kinase cascade as important enrichment pathways. Therefore, DEGs related to signal transduction and MAPK cascade processes under alkali treatment were analyzed, and the results are shown in Figure 7A. DEGs involved in the MAPK cascade and Ca^2+^ and ABA transduction pathways were focused on, including 8 *CPK*/*CDPK*s, 14 *CIPK*s, 3 *CBL*s, 6 *SnRK2*s, 23 *PP2C*s, and 10 *MAPK*s. The results showed that most genes were upregulated after alkali treatment, except *SnRK2*s. The results showed that alkali stress activated ABA and Ca^2+^ signaling in sorghum seedlings, which further promoted the MAPK cascade to promote transcriptional regulation in the plant. Subsequently, we plotted the expression profiles of salt-alkali-related genes *SOS*s, *NHX*s, *HKT*s, and *HAK*s in the SA12h vs. SA0h, SA24h vs. SA0h, and SA72h vs. SA0h comparison groups, as shown in Figure 7B. Although most of the above genes were downregulated in the SA12h vs. SA0h comparison group, they were upregulated in the SA24h vs. SA0h comparison group and the SA72h vs. SA0h comparison group.

## 3. Discussion

In the long-term evolution process, in order to adapt to the saline-alkali environment, plants restore their own osmotic levels and ion homeostasis through a series of changes in physiological metabolism, thereby reducing ion toxicity caused by saline-alkali conditions [18]. Under alkali conditions, the growth and development of crops are retarded, even leading to crop death [10]. In this study, the effects of alkali stress on the growth, photosynthesis, and protective enzymes of sorghum seedlings with different alkali tolerance levels were evaluated from a physiological perspective. The plant height and root length of two different genotypes of sorghum (alkali-sensitive Z1 and alkali-tolerant Z14) showed slow or stagnant growth under alkali stress (80 mM NaHCO_3_) compared with the control group, which indicates that sorghum seedlings of both cultivars experienced large alkali stress, which resulted in severe retardation of their growth. The magnitude of the change was more pronounced with the extension of the treatment time, and the decrease in Z1 was greater than that in Z14.

Reactive oxygen species (ROS) are induced by abiotic stresses and participate in the regulation of many biological processes [19]. Under abiotic stress, plant cells produce a large amount of superoxide anion (O_2_^−^), hydrogen peroxide (H_2_O_2_), hydroxyl radical (-OH), and other substances, which lead to the accumulation of ROS in each organelle, and then destroy the dynamic balance of ROS, leading to oxidative damage and increased permeability of the cell membrane [20]. Malondialdehyde (MDA) is a product of membrane lipid peroxidation [5]. In the present study, we found that the O_2_^−^ and MDA contents of Z1 and Z14 were increased under alkali conditions, and Z1 had higher contents of the above indexes than Z14 at any time point except 0 d. This indicates that the degree of alkali injury to Z1 is severe. Osmoregulatory substances are also important physiological indicators for evaluating plant stress resistance [21]. Proline (Pro) and soluble proteins are classical osmotic regulators that play a key role under salt and saline-alkali stress [22]. In this study, soluble protein and proline showed significant changes. After 3 days of alkali stress, the soluble protein content of Z14 was significantly higher than that of Z1, which indicates that Z14 has a strong osmotic regulation ability under alkali conditions and can help the plant accumulate more solutes and thus maintain a certain osmotic pressure. However, the pro content of Z14 was lower than that of Z1 at the later stage of alkali treatment, which may be caused by the lower expression level of the *P5CS* gene involved in the synthesis of pro at the later stages of stress. Plants can also effectively remove ROS accumulation by enhancing the antioxidant defense system, including SOD, POD, CAT, and APX enzymes [1]. These oxidants can neutralize ROS, effectively alleviate cellular oxidative damage, and maintain intracellular redox balance [23]. A recent study found that the tolerant cultivar Longmu801 had higher SOD, POD, and CAT activities than the sensitive cultivar WL168 in alfalfa under different concentrations of NaCl treatment [24]. This is similar to the results of the present study, in which Z14 was found to have significantly higher levels of antioxidant enzymes than Z1 under alkali treatment. Notably, we also found that Z14-related genes encoding antioxidant enzymes were upregulated under alkali treatment. These results indicated that the expression level of antioxidant enzymes in Z14 was increased under alkali stimulation, and then, the activity of antioxidant enzymes was increased so that the plant could maintain a higher level of antioxidant enzyme activity, more effectively remove the accumulated ROS, and maintain the normal physiological metabolism of cells.

In this study, we used high-throughput transcriptome sequencing to investigate the differential gene expression profile of Z14 in response to alkali. We found that a large number of genes were induced to express at the early stage of alkali treatment (SA12h vs. SA0h). Similar results were found in a study conducted by Sun X and Yang L, who found the highest number of DEGs at the early stage in hosta and arabidopsis under alkali treatments [25,26]. This suggested that the genes in Z14 respond rapidly to alkali stress and gradually adapt to the alkali environment. In addition, transcription factors regulate the expression level of downstream target genes by binding to promoter sequences and play an important role in plant response to stress [27]. Transcription factors, such as *AP2*/*ERF*, *WRKY*, *bHLH*, *NAC*, *MYB,* and *MYB-related* factors, are involved in response to a variety of adverse stresses, such as cold, salt, heat, and drought [28,29,30,31]. In this study, we found that the family with the largest number of transcription factors involved in differential alkaloid stress response in sorghum was the *bHLH* family. In ginger, the expression levels of 11 *bHLH* genes were significantly increased under salt stress, which positively responded to salt stress [32]. The *bHLH* transcription factor *MYC* regulates plant salt tolerance by binding to the 5’UTR region of *P5CS1* and regulating proline synthesis [33]. Other researchers have found that the overexpression of *LcTprxII* can significantly enhance the antioxidant defense ability of plants, thereby improving the alkali tolerance of plants [34]. ERF family members play an important role in plant resistance to alkali stress. Yu et al. found that *GsERF71* improved the alkali tolerance of Arabidopsis by regulating the expression level of H-atpase and enhancing the accumulation of plant auxin [35]. *MYB* proteins can be divided into 2R(R2R3)-*MYB*s, 3R(R1R2R3)-*MYB*s, 4R-*MYB*s, and 1R-*MYB*s (also known as *MYB-related*), which have two, three, four, and one *MYB* repeat sequence, respectively, hence their names [36]. Previous studies have shown that *MYB* transcription factors are involved in the regulation of plant osmotic stress and that *BplMYB46* affects lignin content and cell wall formation by binding to AC-box motifs, allowing plants to survive salt and alkali stresses [37]. In our study, *ERF*, *MYB*, and *MYB-related* transcription factors were significantly induced by alkali stress. This suggests that these TFSs play important roles in alkali stress response in sorghum.

The signal transduction and transduction pathways of plant response to saline-alkali stress are very complex, and the crosstalk between each component regulates plant saline-alkali adaptability [38]. Studies have shown that ABA signal transduction and the MAPK signal pathway are involved in regulating the salt tolerance of *Glaux maritima* [21]. Under salt conditions, *PP2C* upregulates and inhibits the activity of *SnRK2*, promotes the downstream expression of *MAPK17* and *MAPK18*, slows down leaf senescence, and enhances plant salt tolerance [21]. In our study, it was also found that most *PP2C* genes were upregulated and *SnRK2* genes were downregulated under alkali treatment, which in turn activated the expression of *MAPK* genes (*Sobic.009G217500* and *Sobic.001G450500*). These results indicated that the expression of these genes was induced by alkali stress and was involved in plant alkali stress. We also found that genes involved in the Ca^2+^-ion signaling pathway were activated under alkali conditions. Hao et al. also found that exogenous Ca^2+^ addition can effectively increase the expression of genes related to the Ca^2+^ signaling pathway (*CPK*s, *CIPK*s, *CBL*s, etc.), improve ROS levels, and thus enhance salt-alkali tolerance of castor beans [39]. This indicates that signal transduction pathways are activated under alkali stress, which in turn promotes the expression of downstream genes to promote plant metabolism and slow down alkali damage. In addition, many saline-alkali-stress-related genes (two *SOS*s, one *NHX*, two *HKT*s, and two *HAK*s) were upregulated by alkali stress induction in the transcriptome data. This suggests that the expression levels of saline-alkali-related genes are increased under alkali conditions, which maintain intracellular ion homeostasis and thus make plants alkali-tolerant.

**Figure 8:**
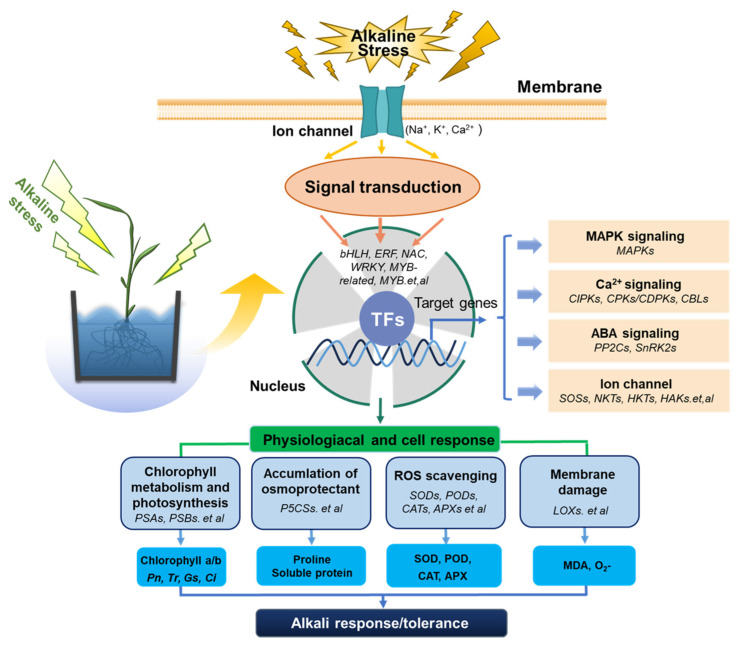
A working model of the response of sorghum seedling leaves to alkali stress.

In summary, we have drawn a working model of sorghum seedling leaves in response to alkali stress (Figure 8). Upon alkali stimulation, Ca^2+^, ABA, and ion signal transduction pathways were activated in sorghum seedlings, which promoted the MAPK cascade. Subsequently, transcription factors are significantly expressed under the influence of upstream kinases, which in turn promote the expression of alkali-stress-related genes, leading to a series of physiological and cellular adaptive responses in sorghum seedlings. These factors include the following: (1) the expression level of genes related to photosynthetic pigment degradation is reduced so that the plant can maintain a certain photosynthetic intensity; (2) accumulation of osmotic substances to maintain the normal osmotic pressure of cells; (3) its own high-level antioxidant enzyme activity, effectively scavenging ROS; (4) the accumulation of membrane damage substances is slow, which reduces the oxidative damage to cells.

## 4. Materials and Methods

### 4.1. Materials and Treatments

In this study, alkali-sensitive material ‘Heilongbuyu180’ (Z1) and alkali-resistant material ‘Heilongbuyu28’ (Z14) were selected as experimental materials. Sorghum seeds were disinfected with 10% sodium hypochlorite and then washed 10 times with distilled water. Subsequently, seeds were germinated at 28 °C for 48 h in an incubator. After 2 days, seeds with the same growth status were selected and transplanted into hydroponic boxes, and the nutrient solution was changed every 3 days using 1/2 Hoagland nutrient solution (pH 6.3–6.8) for hydroponic culture. Sorghum seedlings were grown in a growth chamber with a 16/8 h light/dark cycle at 25 °C. To assess alkali tolerance, 10-day-old seedlings were treated with 80 mM NaHCO_3_, and samples were taken at 0, 1, 3, 5, and 7 d after alkali treatment; 15 plant samples were collected at each treatment time point. The sorghum leaves were frozen in liquid nitrogen and stored at −80 °C for subsequent analysis. Three biological replicates were set for each time point for each index measured.

### 4.2. Determination of Physiological Parameters

To evaluate sorghum seedling growth and physiological responses to alkali, Li-6400XT (Li-6400; LiCor, Huntington Beach, CA, USA) was used to measure gas exchange in sorghum leaves between 9:00 and 11:00. Before the measurement, we subjected the leaf under test to 20 min of light sensing. During measurements, the temperature of the leaf chamber was maintained at 25 ± 1 °C, the relative humidity was 50 ± 5%, and the light intensity was 800 μmol m^−2^ s^−1^. After equilibration to a steady state, the net photosynthetic rate (*Pn*), stomatal conductance (*Gs*), intercellular CO_2_ concentration (*Ci*), and transpiration rate (*Tr*) were measured and recorded in the third fully expanded leaf, with each time point containing five replicates. A chlorophyll fluorometer (FMS-2, Hansatech, UK) was used to determine fluorescence parameters; leaves were acclimated to the dark at 25 °C for 20 min before measurement, and five seedlings were measured at each treatment.

The seedlings were divided into aboveground and belowground parts, and their plant height, root length, and fresh weight were recorded; each index contained three biological replicates per treatment time. Subsequently, all materials were dried at 105 °C for 20 min and oven-dried to a constant weight at 80 °C, and dry weight data were recorded. Chlorophyll and carotenoid contents, protective enzyme activities (SOD, POD, CAT, and APX activities), superoxide anion contents, proline contents, and MDA and soluble protein levels were measured by an ultraviolet spectrophotometer (UV-1500PC; Shanghai Macy, China). Chlorophyll and carotenoids content were calculated (C_a_ = 13.95 × A_665_ − 6.88 × A_649_, C_b_ = 24.96 × A_649_ − 7.32 × A_665_, C_c_ = (1000 × A_470_ − 2.05 × C_a_ − 114.8 × C_b_)/245) [9].

### 4.3. Transcriptome Analysis

The leaves of sorghum seedlings under Z14 alkali stress for 0, 12, 24, and 72 h were sampled. These were named the SA0h control group (SA0h-1, SA0h-2, and SA0h-3), SA12h treatment group (SA12h-1, SA12h-2, and SA12h-3), SA24h treatment group (SA24h-1, SA24h-2, and SA24h-3), and SA72h treatment group (SA72h-1, SA72h-2, and SA72h-3), respectively. Total RNA was isolated from the above 12 samples using a TRIzol reagent (Invitrogen, Carlsbad, CA, USA) according to the manufacturer’s instructions. Libraries were constructed on the basis of good RNA quality and integrity detected using an Agilent 2100 bioanalyzer (Agilent Technologies, Santa Clara, CA, USA). Subsequently, the libraries were sequenced using an Illumina Novaseq 6000 system (Illumina, San Diego, CA, USA).

In order to clearly understand the expression of differentially expressed genes (DEGs) in the leaves of sorghum seedlings under alkali stress, the selection criteria of the ∣log2FC∣ ≥ 1 (FC ≥ 2), SA12h vs. SA0h, SA24h vs. SA0h, SA72h vs. SA0h, SA24h vs. SA12h, SA72h vs. SA12h, and SA72h vs. SA24h DEGs datasets were constructed. In addition, the co-expressed DEGs datasets in the three contrast comparison groups, SA12h vs. SA0h, SA24h vs. SA0h, and SA72h vs. SA0h, were successfully obtained. DEGs were classified using GO (Gene Ontology) and KEGG (Kyoto Encyclopedia of Genes and Genomes) enrichment analysis. The plant TFDB database (http://planttfdb.gao-lab.org/index.php, accessed on 28 May 2025) was used for differentially expressed transcription factor (TF) prediction.

### 4.4. Quantitative Real-Time PCR

To ensure the validity of the transcriptome data, 10 DEGs were randomly selected from the transcriptome for qRT-PCR. RNA was extracted from plant samples as described in Section 4.3, and cDNA was obtained using Vazyme HiScript III RT SuperMix for qPCR (R323-01) kit. The NCBI was used for the design of gene-specific primers; the primer information is shown in Table S5, and the sorghum Actin gene was used as the reference gene. qRT-PCR was performed using the Vazyme ChamQ Universal SYBR qPCR Master Mix kit (Q711) and the real-time detection system, and three replicates were set for each gene. Gene expression levels were calculated by relative quantification (2^−ΔΔCT^).

### 4.5. Statistical Analysis

Excel 2016 was used for data collating, and IBM SPSS Statistics 25 was used for significance analysis. Student’s *t*-test was used for significance analysis, and the symbols *, **, and *** indicate that the differences between different groups were statistically significant (*p* < 0.05, *p* < 0.01, and *p* < 0.001). All data represent the mean (±SD) of three or more biological replicates. GraphPad 10.0, PowerPoint 2016, TBtools-II, and Chiplot (https://www.chiplot.online, accessed on 28 May 2025) were used for the drawing of the pictures.

## 5. Conclusions

This study evaluated the physiological changes and gene expression profiles in different alkali-resistant sorghum varieties under alkali stress. Compared with the alkaline-sensitive Z1, the alkaline-tolerant Z14 maintained higher levels of antioxidant enzyme activities, osmotic regulatory substance content, and chlorophyll and gas exchange and had lower damage indicators (MDA and O_2_^−^). Transcriptome analyses of Z14 under alkali stress showed that 267 DEGs were induced by alkali stress. These results revealed that the MAPK signaling pathway and plant hormone signal transduction and ion transport were important pathways involved in alkali stress. A batch of TFs, including *bHLH*s, *ERF*s, *NAC*s, and *MYB*s, was differentially expressed in response to alkali stress. The transcriptional regulatory network of sorghum under alkali stress was constructed to provide a theoretical basis for the alkali tolerance breeding of sorghum.

## Data Availability

The RNA-seq datasets generated during the current study were submitted to the NCBI Sequence Read Archive under accession number PRJNA1303957.

## Supplementary Materials

The following supporting information can be downloaded at https://www.mdpi.com/article/10.3390/plants14193106/s1. Figure S1. Overall RNA-seq analysis of sequenced samples. Figure S2. qRT-PCR verification of candidate genes obtained by RNA-seq. Figure S3. Overview of DEGs in Z14 sorghum seedlings under 80 mM NaHCO_3_ treatment. Figure S4. Overview of differentially expressed transcription factors under alkali stress. Table S1. Information on RNA-Seq sequencing data of sorghum leaves under alkali stress. Table S2. GO enrichment analysis of 267 co-expressed DEGs. Table S3. KEGG classification of 267 co-expressed DEGs. Table S4. KEGG enrich analysis of 267 co-expressed DEGs. Table S5. Information on 10 pairs of primers used for qRT-PCR verification in this study.

## Abbreviations

The following abbreviations are used in this manuscript: *ΦPSII*, the quantum yield; ABA, abscisic acid; APX, ascorbate peroxidase; CAT, catalase; CBL, calcineurin B-like protein gene; CHL, chlorophyllide a oxygenase; CHLD, magnesium chelatase subunit D; CHLH, magnesium chelatase subunit H; *Ci*, intercellular CO2 concentration; CIPK, calcineurin B-like interacting protein kinase; CPK/CDPK, calcium-dependent protein kinase; CRD, magnesium-protoporphyrin IX monomethyl ester (oxidative) cyclase; DEGs, differentially expressed genes; ETR, electron transport rate; *Fv*/*F_0_*, maximum light energy conversion potential; Fv/Fm, the PSII maximum photochemical efficiency; *Gs*, stomatal conductance; HAK, high-affinity K+ transporter; HEML, glutamate-1-semialdehyde 2,1-aminomutase; HKT, high-affinity K+ transporter 2; LOX, lipoxygenase; MAPK, MAP kinase kinases; MDA, malondialdehyde; NHX, Na+/H+ exchangers; O_2_^−^, superoxide anion; *Pn*, photosynthetic rate; POD, peroxidase; PORA, protochlorophyllide reductase; PP2C, protein phosphatase 2C; PSA, photosystem I; PSB, photosystem II; qRT-PCR, real-time quantitative PCR; ROS, reactive oxygen species; SOD, superoxide dismutase; SOS, salt overly sensitive; SnRK2, sucrose non-fermenting-1-related protein kinase 2; *Tr*, transpiration rate.
