# Supplementary material for: Physiological and Transcriptional Responses of Sorghum Seedlings Under Alkali Stress
Source: Plants (Basel). 2025 Oct 9;14(19):3106. doi: 10.3390/plants14193106 (PMC12526901; doi:10.3390/plants14193106)
Supplement: Supplementary file 1 [file plants-14-03106-s001.zip › plants-3840476-supplementary.pdf]

Table S1. Information of RNA-Seq sequencing data of sorghum leaves under alkali stress.

Sample	Clean Reads (M)	Total Clean Bases	GC Content	%≥Q30
SA0h-1	33,547,192	10,015,095,828	54.19%	92.84%
SA0h-2	34,800,163	10,365,177,792	54.11%	92.57%
SA0h-3	39,526,648	11,772,375,270	53.85%	92.36%
SA12h-1	34,672,639	10,325,717,036	53.63%	93.00%
SA12h-2	33,013,125	9,814,392,960	53.64%	92.92%
SA12h-3	33,233,808	9,921,847,976	52.93%	92.88%
SA24h-1	32,563,942	9,702,153,344	54.49%	92.61%
SA24h-2	22,964,242	6,845,199,750	54.12%	92.96%
SA24h-3	35,738,177	10,620,996,180	54.21%	92.96%
SA72h-1	31,558,032	9,407,641,842	54.05%	91.03%
SA72h-2	31,946,043	9,535,197,496	53.69%	92.11%
SA72h-3	26,967,931	8,052,265,220	54.02%	91.90%

Table S2. GO enrich analysis of 267 co-expressed DEGs.

ID	GeneRatio	Description	Pvalue	Count
GO:0005975	0.068181818	Carbohydrate metabolic process	0.006557142	9
GO:0023014	0.045454545	Signal transduction by protein phosphorylation	1.41355E-06	6
GO:0031098	0.045454545	Stress-activated protein kinase signaling cascade	1.41355E-06	6
GO:0032147	0.045454545	Activation of protein kinase activity	3.94945E-06	6
GO:0023051	0.015151515	Regulation of signaling	0.04125174	2
GO:0010646	0.015151515	Regulation of cell communication	0.045763101	2
GO:0009966	0.015151515	Regulation of signal transduction	0.048083936	2
GO:0044255	0.015151515	Cellular lipid metabolic process	0.048083936	2
GO:0010310	0.007575758	Regulation of hydrogen peroxide metabolic process	0.028241657	1
GO:1901000	0.007575758	Regulation of response to salt stress	0.037478504	1
GO:1902882	0.007575758	Regulation of response to oxidative stress	0.037478504	1
GO:0016021	0.393939394	Integral component of membrane	0.100862055	65
GO:0016020	0.078787879	Membrane	0.274638881	13
GO:0009706	0.006060606	Chloroplast inner membrane	0.239426641	1
GO:0005524	0.174157303	ATP binding	0.048567856	31
GO:0004674	0.06741573	Protein serine/threonine kinase activity	0.019103485	12
GO:0043531	0.04494382	ADP binding	0.01474341	8
GO:0022857	0.028089888	Transmembrane transporter activity	0.142922148	5
GO:0030246	0.016853933	Carbohydrate binding	0.213655062	3
GO:0005975	0.068181818	Carbohydrate metabolic process	0.006557142	9

Table S3. KEGG classification of 267 co-expressed DEGs.

Pathway name	Gene number	Type
Endocytosis	1	Cellular Processes
MAPK signaling pathway - plant	10	Environmental Information Processing
Plant hormone signal transduction	7	Environmental Information Processing
ABC transporters	1	Environmental Information Processing
RNA transport	5	Genetic Information Processing
Protein processing in endoplasmic reticulum	3	Genetic Information Processing
Starch and sucrose metabolism	4	Metabolism
Amino sugar and nucleotide sugar metabolism	3	Metabolism
Glycerophospholipid metabolism	2	Metabolism
Flavone and flavonol biosynthesis	1	Metabolism
Glycolysis / Gluconeogenesis	2	Metabolism
Pentose phosphate pathway	1	Metabolism
Arginine and proline metabolism	1	Metabolism
Carbon metabolism	1	Metabolism
Fatty acid metabolism	1	Metabolism
Fatty acid biosynthesis	1	Metabolism
Fatty acid degradation	1	Metabolism
Fatty acid elongation	1	Metabolism
Glutathione metabolism	1	Metabolism

Table S4. KEGG enrich analysis of 267 co-expressed DEGs.

ID	GeneRatio	Description	Pvalue	Count
ko04016	0.105263158	MAPK signaling pathway-plant	0.030089089	10
ko04075	0.073684211	Plant hormone signal transduction	0.647105644	7
ko00500	0.042105263	Starch and sucrose metabolism	0.531925007	4
ko00051	0.031578947	Fructose and mannose metabolism	0.08101655	3
ko00520	0.031578947	Amino sugar and nucleotide sugar metabolism	0.347690028	3
ko00360	0.021052632	Phenylalanine metabolism	0.180865203	2
ko00010	0.021052632	Glycolysis / Gluconeogenesis	0.586653604	2
ko00564	0.021052632	Glycerophospholipid metabolism	0.60599636	2
ko00061	0.010526316	Fatty acid biosynthesis	0.471633724	1
ko00062	0.010526316	Fatty acid elongation	0.471633724	1
ko00071	0.010526316	Fatty acid degradation	0.507920886	1
ko00944	0.010526316	Flavone and flavonol biosynthesis	0.223259091	1
ko00592	0.010526316	alpha-Linolenic acid metabolism	0.614596646	1
ko01212	0.010526316	Fatty acid metabolism	0.633754013	1
ko03013	0.052631579	RNA transport	0.174807797	5
ko00970	0.042105263	Aminoacyl-tRNA biosynthesis	0.048839116	4
ko03030	0.031578947	DNA replication	0.070180158	3
ko03008	0.021052632	Ribosome biogenesis in eukaryotes	0.43279198	2
ko03440	0.021052632	Homologous recombination	0.436230618	2
ko03440	0.021052632	Homologous recombination	0.436230618	2

Table S5. Information of 10 pairs of primers used for qRT-PCR verification in this study.

Primer name	Primer sequence (5'-3')
Sobic.002G245300-qRT-F	ATCGCAGCTCTCACGATACT
Sobic.002G245300-qRT-R	ACATTTTCGAATCCATGCCA
Sobic.007G219900-qRT-F	ACATGAGCTGAAGTACGTGTATGT
Sobic.007G219900-qRT-R	AGCATCACAGGGCTCGTTTT
Sobic.005G037300-qRT-F	TCGCTATTGCTTACACGGCA
Sobic.005G037300-qRT-R	CGCGGCAGAGAAAATTCCAG
Sobic.009G217500-qRT-F	GCATCGGAGACGACAAGCTA
Sobic.009G217500-qRT-R	CCTCCGATTCAGCTTCCGTT
Sobic.003G268900-qRT-F	ACGCATTTAGGCGTCCATGA
Sobic.003G268900-qRT-R	GCCACGTCAATTAGGAACGG
Sobic.001G143100-qRT-F	CGCTCAGGTGTACAGGTTCG
Sobic.001G143100-qRT-R	GCACCCGTAGGATGGTGTC
Sobic.002G217100-qRT-F	GTACCCCAACTCGTCGATCC
Sobic.002G217100-qRT-R	CTCTCCTTGTGGTAGCTCGC
Sobic.007G122500-qRT-F	AAGAAGAAGCCAGCAGCGAA
Sobic.007G122500-qRT-R	CTCCCCTGCTTTCGATACCC
Sobic.004G089200-qRT-F	TCACAACGCACTCGATGGAA
Sobic.004G089200-qRT-R	GCAGGCTACCATTTGCGATG
Sobic.003G268800-qRT-F	ATGGAACCGTGTACGGATGG
Sobic.003G268800-qRT-R	GGCTTGTTTGAAGTGGAGCG

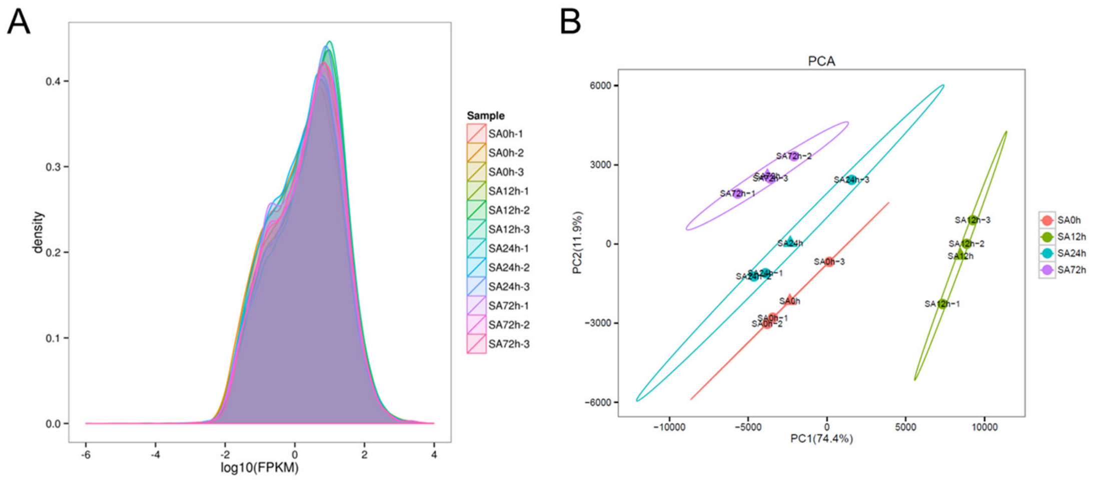

Fig.S1 Overall RNA-seq analysis of sequenced samples.

A. Comparison of FPKM density distribution of each sample. B. Vector plot of two-dimensional scatter plot of PCA.

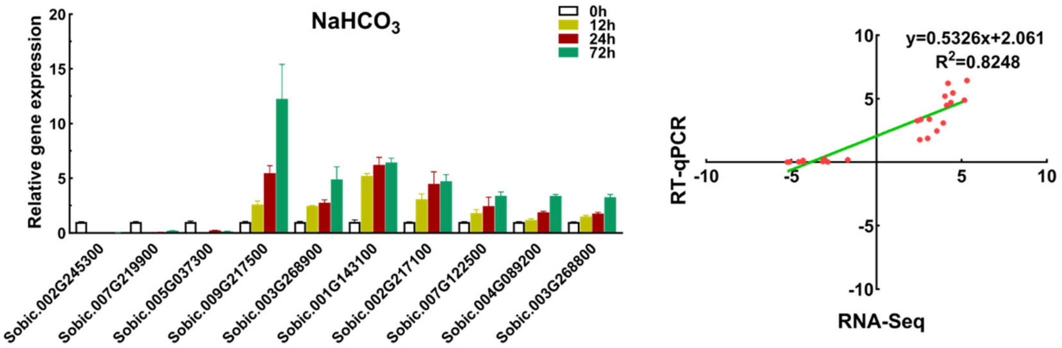

Fig.S2 qRT-PCR verification of candidate genes obtained by RNA-seq.

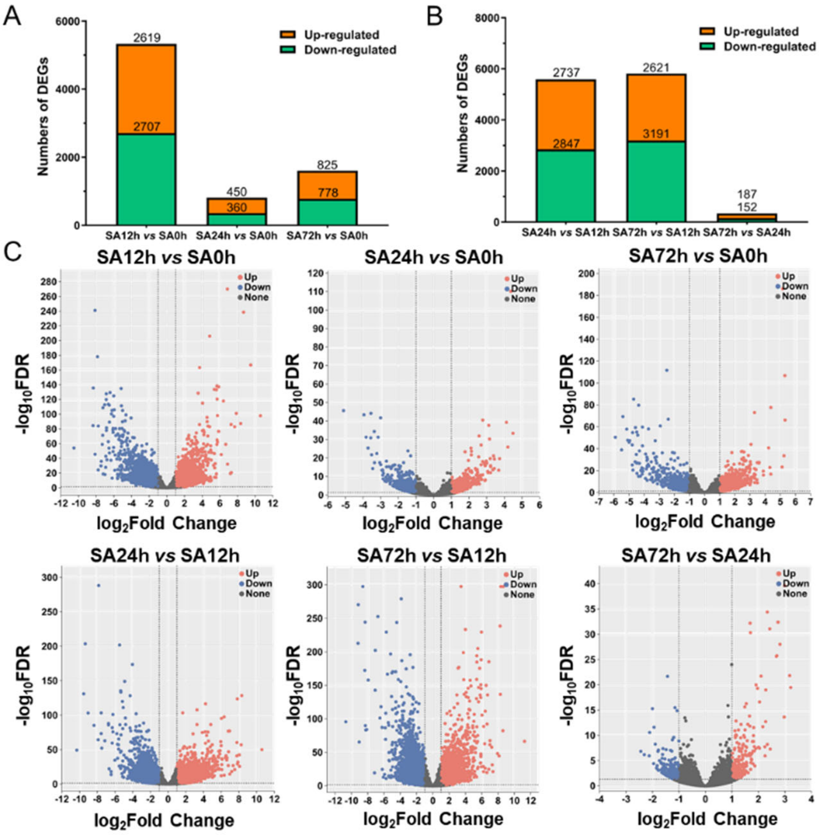

Figure S3. Overview of DEGs in Z14 sorghum seedlings under 80 mM $NaHCO_3$ treatment. (A) DEGs between components aligned with 0 h. (B) DEGs in non-0 h component comparison. (C) Volcano plot of DEGs between components.

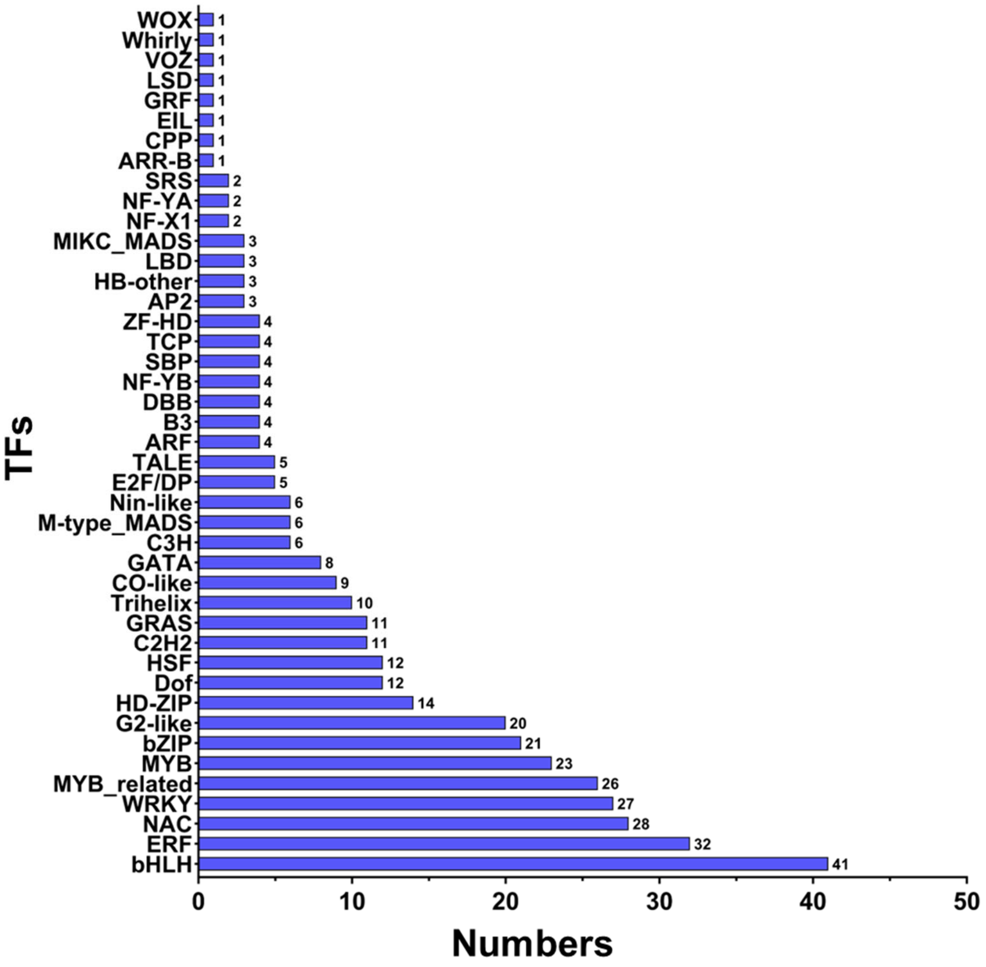

Fig.S4 Overview of differentially expressed transcription factors under alkali stress.
